# Alterations in seminal plasma proteomic profile in men with primary and secondary infertility

**DOI:** 10.1038/s41598-020-64434-1

**Published:** 2020-05-05

**Authors:** Ana D. Martins, Manesh Kumar Panner Selvam, Ashok Agarwal, Marco G. Alves, Saradha Baskaran

**Affiliations:** 1 American Center for Reproductive Medicine, Cleveland Clinic, Cleveland, OH USA; 2 Department of Microscopy, Laboratory of Cell Biology, Institute of Biomedical Sciences Abel Salazar and Unit for Multidisciplinary Research in Biomedicine, University of Porto, Porto, Portugal

**Keywords:** Proteomics, Diagnostic markers

## Abstract

Proteome of seminal plasma provides profound information related to the male reproductive health. This pilot study was conducted to characterize proteomic profile of seminal plasma from men with primary, or secondary infertility and compare it with proven fertile men. Study participants (n = 59) were recruited at the Cleveland Clinic and divided according to their fertility status: proven fertile (n = 39); primary infertility (n = 11) and secondary infertility (n = 9). Proteomic shotgun analysis revealed a total of 515 peptides common to primary infertility and control group; whereas 523 peptides were common to secondary infertility and control group. Bioinformatic analysis revealed dysregulation of biological processes such as cell secretion and vesicle mediated transport in primary infertility, whereas immune system response, regulation of proteolysis and iron homeostasis were dysregulated in secondary infertility. Western blot validation showed overexpression of ANXA2 and CDC42, and underexpression of SEMG2 proteins in primary infertility; and overexpression of ANXA2 and APP proteins in secondary infertility. This study elucidates the potential role of differentially expressed proteins in the seminal plasma as diagnostic biomarker for primary and secondary infertility. Furthermore, our results suggest maturation failure and immune reaction response as the main cause of infertility in men with primary and secondary infertility, respectively. Additional validation of the proteins involved in the above pathways is warranted.

## Introduction

Infertility globally affects 15% of couples and is now classified as a disease of the reproductive system by the World Health Organization (WHO)^1^. Based on the presence or absence of previous successful pregnancies, infertility can be divided as primary and secondary. Couples who were unable to become pregnant after at least 1 year of sexual intercourse without contraceptive methods are referred as primary infertility. On the other hand, couples who were able to get pregnant at least once, but not subsequently are referred as secondary infertility. Prevalence of primary infertility (1.5 to 2.6%) is reported to be lower than secondary infertility (7.2 to 18%)^2^. Approximately, 50% of all reported couple infertility cases can be attributed to the male factor^3,4^ though the reasons remain unknown. Basic semen analysis is one of the first steps in the evaluation of male infertility. This analysis provides both macroscopic (volume, pH, color, viscosity) and microscopic characteristics (sperm concentration, total motility, progressive motility, sperm morphology) of semen^5^. The semen analysis remains the cornerstone of male fertility evaluation. However, it does not provide a systematic explanation for the subcellular changes that occur in the spermatozoa of infertile men, which necessitates a more in-depth analysis and understanding at molecular level^6,7^.

Spermatozoa acquires fertilizing potential during their epididymal maturation phase before ejaculation^8^. The ejaculated semen contains both cellular (spermatozoa) and non-cellular (seminal plasma) components. The seminal plasma is composed of secretions from testis, epididymis, prostate, seminal vesicles and bulbo-urethral glands;^9,10^ it provides nourishment and protection to spermatozoa^11,12^. It also plays a crucial role in sperm maturation, capacitation, acrosome reaction and fertilization^11,12^. Composition of the seminal plasma protein and their interaction with sperm surface influence the fertilizing capacity of spermatozoa^12^.

In recent years, there is an increased number of reports on seminal plasma proteome to identify potential biomarkers for different pathologies and conditions related to infertility. This includes varicocele^13–16^, oxidative stress mediated male infertility^17–20^, nonobstructive azoospermia^21–23^, asthenozoospermia^24,25^, oligoasthenozoospermia^26^, secondary hypogonadism^27^ and prostate cancer^19,28,29^. Borrachina and collaborators performed a proteomic study in the seminal plasma of infertile patients with normozoospermia, azoospermia, asthenozoospermia and oligoasthenozoospermia and concluded that the current classification of infertile patients based on altered semen parameters resulted in a high heterogeneity in the seminal plasma proteomic profile^30^. Agarwal and collaborators^17^ performed proteomic analysis of seminal plasma of infertile men having high levels of seminal reactive oxygen species (ROS) and compared it with proven fertile men with normal ROS in semen. Utilizing proteomic and bioinformatic analysis, it has been suggested that membrane metallo-endopeptidase (MME) and family with sequence similarity 3 (FAM3D) along with ROS levels in the seminal plasma can serve as good markers for diagnosis of male infertility^17^. Seminal plasma proteomic study in idiopathic oligoasthenozoospermic men revealed differential expression of proteins such as glycosylated epidydimal secretory protein E1(NPC2), galectin-3-binding protein (M2BP) or lipocalin-1 which provides a basis for further investigations of mechanisms underlying oligoasthenozoospermia^26^. These studies provided important information related to mechanisms associated with male infertility, however did not provide any evidence on the seminal plasma proteomics based on the type of infertility.

The present study was conducted with the following objectives: 1) to profile the seminal plasma proteome of primary and secondary infertile men compared to men with proven fertility, 2) to identify the differentially expressed proteins (DEPs) that could serve as potential biomarkers for primary and secondary infertility.

## Materials and Methods

### Study subject’s selection

This pilot study (IRB #11–451) was approved by the Institutional Review Board (IRB) of Cleveland Clinic. All the subjects (27–52 years) enrolled in this study signed an informed written consent. Semen samples were obtained from 39 healthy male donors (control group) who had fathered a child in the past 2 years; 11 patients with primary infertility (primary infertility group) and 9 patients with secondary infertility (secondary infertility group). The individuals from the control group had normal semen parameters according to the WHO 2010 guidelines^1^. All the methods were performed in accordance with the relevant guidelines and regulations according to the declaration of Helsinki (https://www.wma.net/policies-post/wma-declaration-of-helsinki-ethical-principles-for-medical-research-involving-human-subjects/).

### Inclusion and exclusion criteria

All subjects enrolled in this study were non-smokers and had never exposed to harmful radiations or environmental stress. Men with azoospermia, oligozoospermia and leukocytospermia were excluded from the study. Furthermore, men under the supportive medication, steroids or drugs were excluded from the study. Additionally, patients with systemic reproductive tract inflammation, genetic defects and sexually transmitted disease were also excluded.

### Semen analysis

Semen samples were collected at the Andrology Laboratory by masturbation after sexual abstinence of least 2–7 days. Samples were allowed to liquefy completely for 20 minutes at 37 °C, and semen analysis was performed according to the WHO (2010) guidelines^31^ using a disposable Leja sperm counting chamber (Spectrum Technologies, Healdsburg, CA) to evaluate sperm count, motility and round cells. After routine semen analysis, the left-over samples were centrifuged for 7 minutes at 1000 × g. The clear seminal plasma was aspirated from the samples and stored at −80 °C for proteomic studies.

### Sample preparation for proteomic analysis

The samples used for proteomic analysis were in compliance with the Minimum Information about a Proteomics Experiment (MIAPE) guidelines of the Human Proteome Organization’s Proteomics Standards Initiative (HUPO-PSI) for reporting proteomics studies^32^. Seminal plasma samples were thawed at room temperature and centrifuged at 3000 × g for 30 minutes to remove any contamination with spermatozoa or other cellular debris. The samples were mixed (1:1 ratio) with the proteinase inhibitor cocktail (Roche, Indianapolis, IN) prepared in phosphate buffer saline (PBS) to prevent proteolysis during sample handling. The protein concentration was determined using a commercial kit, bicinchoninic acid (BCA) kit (Thermo, Rockford, IL), following the manufacturer instructions.

Pooled samples from control (n = 3); primary infertility (n = 3) and secondary infertility (n = 3) were used for proteomic analysis. Equal concentration of proteins from each individual sample was used to normalize the protein concentration in each group. Sample normalization was done by pooling the samples to overcome the biological variation^33,34^. The samples were mixed with SDS-PAGE buffer and subjected to 1D-PAGE in triplicate runs to overcome the technical variation. After electrophoresis, each gel was cut into 6 pieces, digested with 5 μL trypsin (10 ng/μL), 50 mM ammonium bicarbonate, and incubated overnight at room temperature. Prior to in-gel digestion, the samples (cut lanes) were alkylated with iodoacetamide and reduced with dithiothreitol. The peptides from the digested gel were extracted in two aliquots of 30 μL acetonitrile (10%) with formic acid (5%). The two aliquots were pooled together and evaporated to <10 μL and then diluted with 1% acetic acid to make up a final volume to 30 μL.

### Liquid chromatography-tandem mass spectrometry analysis (LC-MS/MS)

Proteomic profiling of seminal plasma was carried out using a Finnigan LTQ linear ion trap mass spectrometer LC-MS/MS system. The peptides were fractionated by injecting 5 μL into a high-performance liquid chromatography (HPLC) column (Phenomenex Jupiter C18 reversed-phase capillary chromatography column). Fractions containing the peptides were eluted in acetonitrile/0.1% formic acid at a flow rate of 0.25 μL/min and were introduced into the source of the mass spectrometer on-line. The micro-electrospray ion source was operated at 2.5 kV. A full spectral scan was performed by utilizing the data dependent multitask ability of the instrument to determine peptide molecular weights and amino acid sequence of the peptides^35^.

### Database searching and protein identification

Tandem mass spectra generated by LC-MS/MS system were retrieved using Proteome Discoverer version 1.4.1.288(https://www.thermofisher.com/us/en/home/industrial/mass-spectrometry/liquid-chromatography-mass-spectrometry-lc-ms/lc-ms-software/multi-omics-data-analysis/proteome-discoverer-software.html). Mascot (Matrix Science, London, UK; version 2.3.02), Sequest (Thermo Fisher Scientific, San Jose, CA, USA; version 1.4.0.288) and X! Tandem (The GPM, thegpm.org; version CYCLONE (2010.12.01.1) search was performed on all the MS/MS raw files. The search was limited to the human reference sequences database (http://www.hprd.org/) assuming the digestion enzyme trypsin. The mass tolerance for parent ion was set to 10 parts per million (ppm) and for fragment ion 1.0 Da. The search results were uploaded into the Scaffold (version 4.0.6.1; Proteome Software Inc., Portland, OR) program as previously described^36^. Protein probabilities were assigned by the Protein Prophet (Systems Biology, Seattle, WA) algorithm. Annotation of proteins was performed using Gene Ontology (GO) terms from National Center for Biotechnology Information (NCBI).

### Quantitative proteomics

The relative quantification of the proteins was performed by comparing the number of spectra, termed spectral counts (SpCs) in control vs primary infertility group and control vs. secondary infertility group. To achieve the false detection rate (FDR) < 1%, protein identification criteria was established at >99% probability as explained in our previous study^37^. The abundance of the proteins was determined by matching the SpCs and classified as high (H), medium (M), low (L), or very low (VL). To overcome the sample-to-sample variation, normalization of spectral counts was done using the normalized spectral abundance factor (NSAF). In general, longer proteins have more peptide identifications than shorter proteins. NSAF ratio determines the actual expression of the protein in the samples, proteins with ratio <1 and >1 are considered underexpressed and overexpressed, respectively. Different constraints for fold-change cut-offs and significance tests (P value) based on the average SpCs from 3 replicate runs were applied to obtain the DEPs^36^. Appropriate filters were applied to minimize the errors due to the presence of low abundance proteins. Abundance and the expression of DEPs are based on the following criteria: (i) VL - SpC range, 1.7–7; NSAF ratio (≥2.5 for upregulated and ≤0.4 for downregulated proteins); and P ≤ 0.001, (ii) L - SpC range, 8–19; NSAF ratio (≥2.5 for upregulated and ≤0.4 for downregulated proteins); and P ≤ 0.01, (iii) M - SpC range, between 20 and 79; NSAF ratio (≥2.0 for upregulated and ≤0.5 for downregulated proteins); and P ≤ 0.05, (iv) H - SpC, >80; NSAF ratio (≥1.5 for upregulated and ≤0.67 for downregulated proteins); and P ≤ 0.05.

### Bioinformatic analysis

DEPs identified in the control vs primary infertility group and control vs secondary infertility group were subjected to functional annotation and enrichment analysis using both, publicly available bioinformatic annotation tools and databases such as GO Term Finder, GO Term Mapper, UniProt, and Database for Annotation, Visualization and Integrated Discovery (DAVID) (http://david.niaid.nih.gov). Search Tool for the Retrieval of Interacting Genes/Proteins (STRING) analysis was performed using the online tool to identify protein-protein interaction networks (https://string-db.org/). Proprietary software package Metacore (GeneGo Inc.) was also used to identify the upstream regulators involved in the enriched pathways.

### Protein validation by western blot and total protein staining

The key proteins involved in reproductive functions and fertilization process were selected for validation. The functions of these proteins are well described in the literature. Based on function and role of proteins related to fertility potential, six DEPs from primary infertility and five DEPs from secondary infertility were selected for validation. The DEPs were validated by western blot (WB) in individual samples from the control group (n = 6) and primary (n = 6) or secondary (n = 6) infertility group. A total of 20 µg of protein per sample was mixed with equal volume of loading buffer (125 mM Tris-HCl, pH 6.8, 2% SDS, 5% glycerol, 0.003% bromophenol blue, and 1% β-mercaptoethanol). The sample mixture was boiled for 10 minutes and kept on the ice for 5 minutes. 30 µL of each sample was loaded into a 4%–15% SDS–polyacrylamide gel and electrophoresed for 2 h at 90 V along with a set of molecular weight marker (Sigma Chemical Co., St. Louis, MO, USA). The resolved protein bands were then transferred onto polyvinylidene difluoride (PVDF) membranes at 20 V for 30 minutes using a transfer buffer (25 mM Tris base, 192 mM glycine, and 20% methanol). PVDF membranes were blocked with Tris-buffered saline-Tween-20 (TBST) with 5% bovine serum albumin (BSA) and used for immunodetection of seminal plasma proteins^38^. For each protein analysis, specific primary antibodies were incubated overnight at 4 °C overnight (Table 1). Subsequently, the membranes were washed four times with TBST for 10 minutes and incubated with the secondary antibodies at room temperature for 1 h (Table 1). The same membranes were washed four times with TBST (10 minutes) and finally treated with enhanced chemiluminescence (ECL) reagent (GE Healthcare, Marlborough, USA) for 5 minutes. ECL reacted blots were exposed to Chemi-Doc (ChemiDoc MP Imaging System, Bio-Rad, Hercules, USA) to detect the chemiluminescence signals^38^.

**Table 1:** List of the primary and secondary antibodies used in this study.

Primary				Secondary		
Protein	Manufacturer	Source	Dilution	Antibody	Manufacturer	Dilution
Annexin A2	ab54771	Mouse	1:1000	Anti-Mouse Rabbit IgG	ab6728	1:10000
CD63	ab118307	Rabbit	1:500			
CDC42	ab64533		1:1000			
PRDX2	ab71533		1:1000			
SEMG1	sc34719		1:100	Anti-Rabbit Goat IgG	ab97051	
SEMG2	ab108085		1:1000			
APP	ab32136		1:5000			
C4	ab173577		1:1000			

APP - Amyloid Precursor Protein; PRDX2 - Peroxiredoxin-2; SEMG – Semenogelin.

The total amount of protein present in the membranes were quantified using a Colloidal Gold Total Protein Stain (Bio-Rad, Hercules, USA). The protocol was performed according to manufacturer instructions. Briefly, the membranes were washed twice for 10 minutes in distilled water and stained with total colloidal gold protein stain by gentle shaking for 2 h at room temperature. Stained membranes were washed twice with distilled water for 10 minutes, and the densitometry image was captured using colorimetric mode on Chemi-Doc (ChemiDoc MP Imaging System, Bio-Rad, Hercules, USA)^38^.

### Statistical analysis

MedCalc Statistical Software (V. 17.8; MedCalc Software, Ostend, Belgium) (https://www.medcalc.org/) was used for data analysis. Mann-Whitney test was performed to compare (control vs. primary infertility group and control vs. secondary infertility group) the semen parameters and the expression levels of the proteins validated using WB technique. The results were considered significant with P < 0.05.

## Results

### Semen parameters of men with primary infertility and men with secondary infertility

Semen analysis showed significant decrease (P < 0.05) in sperm concentration, motility, and total count, and total motile sperm count in both primary infertility and secondary infertility group compared to control group (Table 2).

**Table 2:** Semen parameters in control, primary and secondary infertility groups.

Group	Volume ± SD (ml)	Concentration ± SD (x10^6^ /ml)	Motility ± SD (%)	Total Count ± SD (x10^6^)	Total Motile Sperm ± SD (x10^6^)
Control	3.67 ± 1.97	76.48 ± 37.34	60 ± 10.95	273.37 ± 180.20	163.39 ± 109.10
Primary Infertility *P-value*	3.70 ± 4.06	30.48 ± 39.02	37 ± 21.47	119.52 ± 171.3	22.59 ± 70.87
	0.251	0.003	0.002	0.007	0.006
Secondary Infertility *P-value*	3.84 ± 1.85	39.25 ± 34.52	48 ± 13.35	166.05 ± 183.80	91.49 ± 106.60
	0.711	0.009	0.021	0.056	0.029

### Seminal plasma proteome of primary and secondary infertility

**Figure 1:**
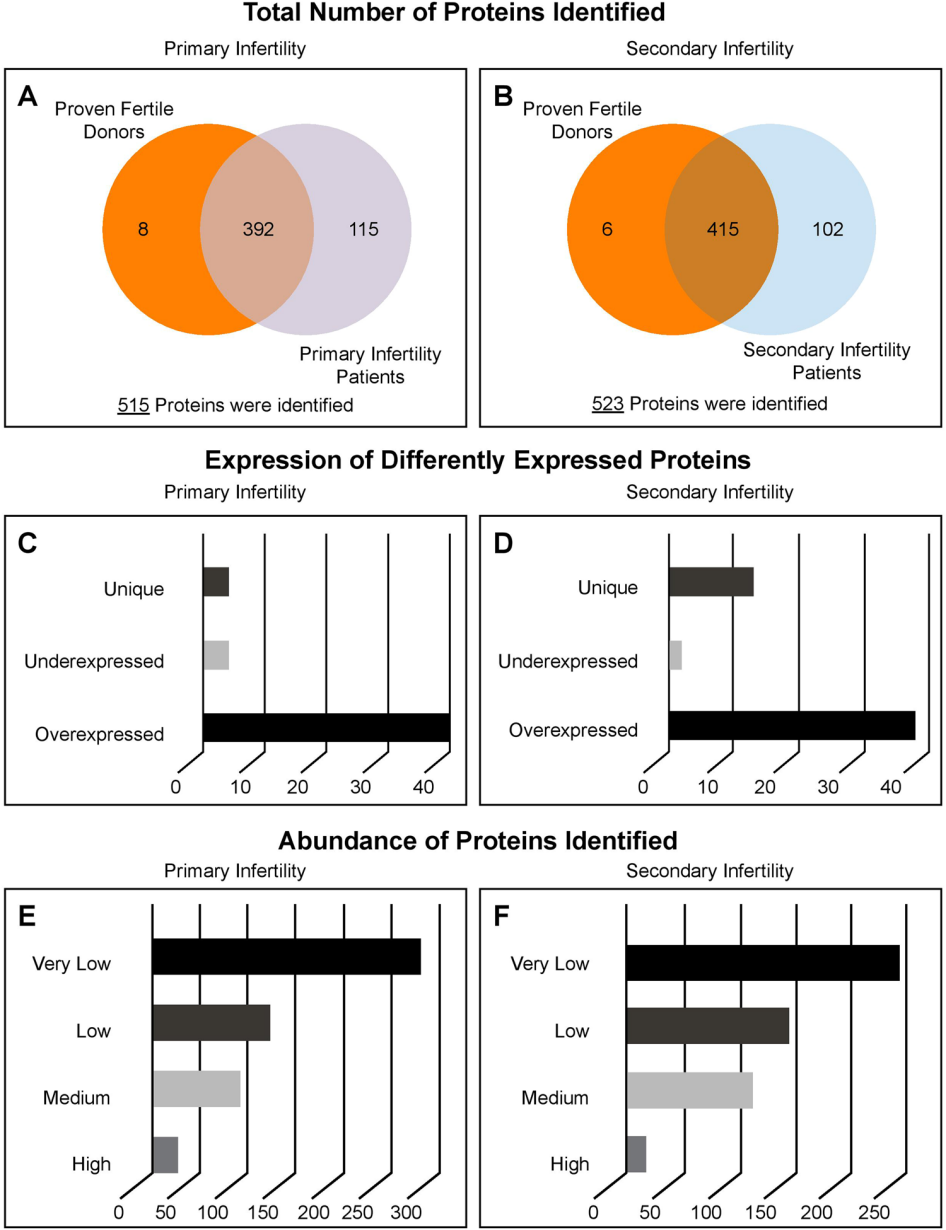
Total number proteins identified in the seminal of proven fertile donors’ group and patients with primary (**A**) and secondary infertility (**B**) by LC-MS/MS spectrometry. Differentially expressed proteins of experimental groups (**C,D**). Distribution of the identified proteins based on their relative abundance (**E,F**).

LC-MS/ MS analysis identified a total of 515 different peptides in primary infertility and control group. Out of these, 392 peptides were common to primary infertility and control group, 8 were unique to control group and 115 were unique to primary infertility group (Fig. 1A). Relative abundance of identified peptides revealed 265 peptides with very low, 126 had low, 91 had medium and 25 had high relatively abundance (Fig. 1E). Only 48 seminal plasma proteins were identified as DEPs between primary infertility and control group (Table 3). Of the 48 identified DEPs, 40 were overexpressed, 4 were underexpressed and 4 were unique to primary infertility group compared with control group (Fig. 1C).

**Table 3:** Differently expressed proteins identified in seminal plasma from men with primary infertility compared with control men.

Protein	Accession number	Average SC		Abundance		NSAF ratio	T-Test	Expression
		Control	PI	Control	PI	PI/ Control	P-value	
Semenogelin-1 preproprotein	4506883	698.3	312.0	H	H	0.28	0.001	UE
Semenogelin-2 precursor	4506885	1321.3	598.0	H	H	0.30	<0.001	UE
Extracellular matrix protein 1 isoform 3 precursor	322302700	66.0	80.3	H	H	0.63	0.001	UE
Prolactin-inducible protein precursor	4505821	258.3	277.3	H	H	0.66	0.002	UE
Actin. Cytoplasmic 2	316659409	53.7	113.3	M	H	1.72	0.004	OE
Triosephosphate isomerase isoform 2	226529917	15.0	43.7	L	M	2.08	0.012	OE
Sorbitol dehydrogenase	156627571	19.7	47.3	L	M	2.10	0.008	OE
Transmembrane protease serine 2 isoform 1	227499990	8.0	23.0	L	M	2.13	0.001	OE
Neprilysin isoform X1	578807443	15.0	63.7	L	M	2.18	0.008	OE
Phosphoglycerate kinase 1	4505763	9.3	25.0	L	M	2.19	0.002	OE
Prostaglandin-H2 D-isomerase precursor	32171249	14.0	37.0	L	M	2.19	0.013	OE
Serotransferrin precursor	4557871	63.3	282.0	M	H	2.24	<0.001	OE
Peroxiredoxin-2	32189392	11.3	35.7	L	M	2.34	0.026	OE
Sialate O-acetylesterase isoform 1 precursor	24850115	6.7	28.0	VL	M	2.38	0.034	OE
L-lactate dehydrogenase B chain	291575128	9.0	23.7	L	M	2.39	0.011	OE
Iggfc-binding protein precursor	154146262	16.3	54.0	L	M	2.41	0.004	OE
Serpin B6 isoform a	41152086	8.0	23.7	L	M	2.41	0.004	OE
Chloride intracellular channel protein 1	14251209	3.3	11.7	VL	L	2.50	0.001	OE
Ras-related protein Rab-3B	19923750	11.0	39.0	L	M	2.77	0.012	OE
Creatine kinase B-type	21536286	15.7	57.3	L	M	2.80	0.005	OE
Di-N-acetylchitobiase precursor	4758092	3.0	11.7	VL	L	3.28	0.003	OE
CD63 antigen isoform A	383872447	3.0	10.3	VL	L	3.30	0.005	OE
Annexin A1	4502101	7.7	34.3	VL	M	3.37	0.015	OE
L-lactate dehydrogenase A chain isoform 1	5031857	5.0	19.3	VL	L	3.37	0.010	OE
Cytosolic non-specific dipeptidase isoform X2	530414265	8.7	37.3	L	M	3.47	0.011	OE
Receptor-type tyrosine-protein phosphatase S isoform X7	530425347	5.0	25.0	VL	M	3.66	0.003	OE
Annexin A2 isoform 2	50845386	4.7	22.0	VL	M	3.86	0.016	OE
Ras-related protein Rab-27B isoform X1	530414276	2.0	10.0	VL	L	3.87	0.009	OE
Protein-glutamine gamma-glutamyltransferase 4	156627577	31.3	255.3	L	H	4.10	<0.001	OE
Collagen alpha-1(XVIII) chain isoform 2 precursor	110611233	1.3	9.0	VL	L	4.35	0.001	OE
Plastin-2 isoform X2	530402335	10.0	62.0	VL	M	4.46	0.027	OE
Desmocollin-1 isoform Dsc1a preproprotein	13435361	2.0	11.3	VL	L	5.08	0.002	OE
Laminin subunit gamma-1 precursor	145309326	2.0	19.7	VL	L	5.26	0.006	OE
Ras-related protein Rab-27A	19923264	3.0	20.7	VL	M	5.58	0.001	OE
Dipeptidase 3 isoform a precursor	193211608	0.7	9.3	VL	L	6.84	0.009	OE
Desmoplakin isoform I	58530840	9.7	56.3	L	M	7.52	0.048	OE
Agrin precursor	54873613	1.3	15.0	VL	L	7.72	0.005	OE
Amiloride-sensitive amine oxidase [copper-containing] isoform X3	578814090	2.7	40.7	VL	M	7.94	0.009	OE
Laminin subunit alpha-5 isoform X1	578835999	1.7	27.0	VL	M	8.95	0.001	OE
Lactoylglutathione lyase	118402586	1.3	13.7	VL	L	8.96	0.001	OE
Golgi apparatus protein 1 isoform 2 precursor	224586815	0.7	8.7	VL	L	11.35	0.002	OE
Choline transporter-like protein 4 isoform 1	148612887	0.3	8.7	VL	L	12.95	0.001	OE
Junction plakoglobin isoform X1	530412116	2.0	32.7	VL	M	21.31	0.010	OE
Polymeric immunoglobulin receptor isoform X1	530366266	0.3	24.0	VL	M	41.94	0.001	OE
Cell division control protein 42 homolog isoform 1 precursor	4757952	0.0	3.3	—	VL	—	<0.001	Unique
Glyoxalase domain-containing protein 4	217330598	0.0	2.3	—	VL	—	<0.001	Unique
Ferritin heavy chain	56682959	0.0	4.0	—	VL	—	<0.001	Unique
Importin-5 isoform X2	530423350	0.0	5.0	—	VL	—	0.001	Unique

Abbreviations: SC - Spectral counts; NSAF - Normalized spectral abundance factor; H - High; M - Medium; L - Low; VL - Very Low; UE - Underexpressed; OE – Overexpressed; PI- Primary Infertility.

The analysis identified a total of 523 different peptides in secondary infertility and control group. Of these, 415 peptides were common to secondary infertility and control group, 6 were unique to control group and 102 were unique to secondary infertility group (Fig. 1B). Relative abundance of the identified proteins revealed 245 proteins with very low abundance, 143 with low abundance, 110 with medium abundance and 15 proteins with high relative abundance (Fig. 1F). A total of 53 seminal plasma proteins were found to be differentially expressed between secondary infertility and control group (Table 4). Of these 53 DEPs, 2 were underexpressed, 38 were overexpressed in secondary infertility group compared with control group, and 13 DEPs were unique to secondary infertility group (Fig. 1D).

**Table 4:** Differently expressed proteins identified in seminal plasma from men with secondary infertility compared with control men.

Protein	Accession Number	Average SC		Abundance		NSAF ratio	T-Test	Expression
		Control	SI	Control	SI	SI/ Control	*P-value*	
Semenogelin-2 precursor	4506885	1321.3	1310.0	H	H	0.54	0.001	UE
Semenogelin-1 preproprotein	4506883	698.7	742.0	H	H	0.59	0.010	UE
Serotransferrin precursor	4557871	63.3	214.3	M	H	1.52	0.010	OE
Alpha-1-antichymotrypsin precursor	50659080	40.7	144.0	M	H	1.90	0.001	OE
Elongation factor 1-alpha 1	4503471	20.3	56.7	M	M	2.02	0.000	OE
78 glucose-regulated protein precursor	16507237	8.7	44.3	L	M	2.24	0.048	OE
Tubulin beta-4B chain	5174735	13.7	44.0	L	M	2.27	0.011	OE
Creatine kinase B-type	21536286	15.7	53.0	L	M	2.29	0.002	OE
Cytosolic non-specific dipeptidase isoform X2	530414265	8.7	32.0	L	M	2.37	0.040	OE
Transmembrane protease serine 2 isoform 2	205360943	8.0	34.0	L	M	2.46	0.004	OE
Annexin A1	4502101	7.7	33.0	VL	M	2.75	0.013	OE
Kallistatin isoform 2 precursor	21361302	9.0	41.0	L	M	2.75	0.026	OE
Purine nucleoside phosphorylase	157168362	4.7	24.0	VL	M	2.79	0.011	OE
Alpha-2-antiplasmin isoform X1	578840157	11.0	46.0	L	M	2.90	0.017	OE
Receptor-type tyrosine-protein phosphatase S isoform X1	530425335	5.0	25.3	VL	M	2.92	0.019	OE
Annexin A2 isoform 1	50845388	4.7	20.0	VL	M	3.06	0.018	OE
Cullin-associated NEDD8-dissociated protein 1	21361794	4.3	26.3	VL	M	3.10	0.021	OE
Complement C4-A isoform 1 preproprotein	67190748	7.3	49.7	VL	M	3.19	0.032	OE
L-lactate dehydrogenase A chain isoform 1	5031857	5.0	25.3	VL	M	3.25	0.001	OE
Ras-related protein Rab-27A isoform X2	530406261	3.0	18.0	VL	L	3.28	0.005	OE
Beta-hexosaminidase subunit alpha preproprotein	189181666	6.3	34.0	VL	M	3.50	0.002	OE
Ezrin	21614499	2.0	9.7	VL	L	3.50	0.004	OE
Olfactomedin-4 precursor	32313593	3.0	25.0	VL	M	3.53	0.000	OE
Beta-galactosidase isoform b	119372312	3.0	22.0	VL	M	3.57	0.003	OE
Tubulin alpha-1C chain	14389309	9.0	40.3	L	M	3.60	0.001	OE
Maltase-glucoamylase. intestinal isoform X1	578814724	3.3	29.0	VL	M	3.69	0.010	OE
Heat shock cognate 71 protein isoform X1	578822169	3.0	27.7	VL	M	4.02	0.003	OE
Plastin-2 isoform X2	530402335	10.0	65.7	L	M	4.11	0.006	OE
Legumain preproprotein	56682962	3.0	21.0	VL	M	4.80	0.004	OE
Alpha-1-acid glycoprotein 2 precursor	4505529	1.7	13.0	VL	L	4.87	0.006	OE
Lactoylglutathione lyase	118402586	1.3	11.0	VL	L	5.48	0.002	OE
Alpha-1B-glycoprotein precursor	21071030	1.3	17.0	VL	L	6.11	0.003	OE
Laminin subunit alpha-5 isoform X1	578835999	1.7	27.0	VL	M	7.06	0.022	OE
Lipocalin-15 precursor	42714611	0.7	10.0	VL	L	8.39	0.001	OE
Dipeptidase 3 isoform a precursor	193211608	0.7	13.3	VL	L	10.10	0.002	OE
Complement C3 precursor	115298678	2.7	92.0	VL	H	15.37	0.004	OE
Programmed cell death 6-interacting protein isoform 1	22027538	0.7	21.0	VL	M	15.60	0.026	OE
Adenylyl cyclase-associated protein 1	5453595	0.3	11.3	VL	L	22.01	0.002	OE
Polymeric immunoglobulin receptor isoform X1	530366266	0.3	29.0	VL	M	45.00	0.004	OE
Histone H2A type 2-A	4504251	0.3	18.0	VL	L	49.22	0.005	OE
Ferritin heavy chain	56682959	0.0	2.0	—	VL	—	0.000	Unique
Fructose-1.6-bisphosphatase 1	16579888	0.0	6.7	—	VL	—	0.001	Unique
Nephronectin isoform A precursor	296011067	0.0	6.0	—	VL	—	0.000	Unique
Transforming protein rhoa precursor	10835049	0.0	2.0	—	VL	—	0.000	Unique
Ceruloplasmin precursor	4557485	0.0	9.3	—	L	—	0.000	Unique
Importin-5 isoform X2	530423350	0.0	12.0	—	L	—	0.000	Unique
Lysosomal Pro-X carboxypeptidase isoform 1 preproprotein	4826940	0.0	10.3	—	L	—	0.000	Unique
Heat shock 70 protein 1-like isoform X1	530381921	0.0	8.7	—	L	—	0.000	Unique
Carboxylesterase 5 A isoform 1 precursor	219521907	0.0	14.3	—	L	—	0.001	Unique
Kunitz-type protease inhibitor 1 isoform 1 precursor	32313599	0.0	8.0	—	L	—	0.004	Unique
Myosin-9	12667788	0.0	17.0	—	L	—	0.005	Unique
Amyloid beta A4 protein isoform f precursor	209915573	0.0	8.3	—	L	—	0.007	Unique
Alpha-crystallin A chain-like isoform X1	578836360	0.0	51.3	—	M	—	0.045	Unique

Abbreviations: SC - Spectral counts; NSAF - Normalized spectral abundance factor; H - High; M - Medium; L - Low; VL - Very Low; UE - Underexpressed; OE – Overexpressed; SI – Secondary Infertility.

### Bioinformatic analysis of seminal plasma DEPs in men with primary and secondary infertility

**Figure 2:**
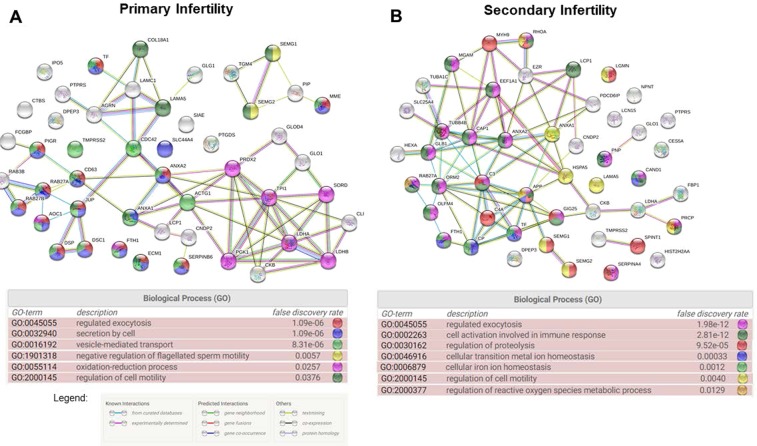
String network and Biological processes identified in differently expressed proteins of seminal plasma of men with primary infertility (**A**) and men with secondary infertility (**B**) compared with proven fertile donors’ group.

**Figure 3:**
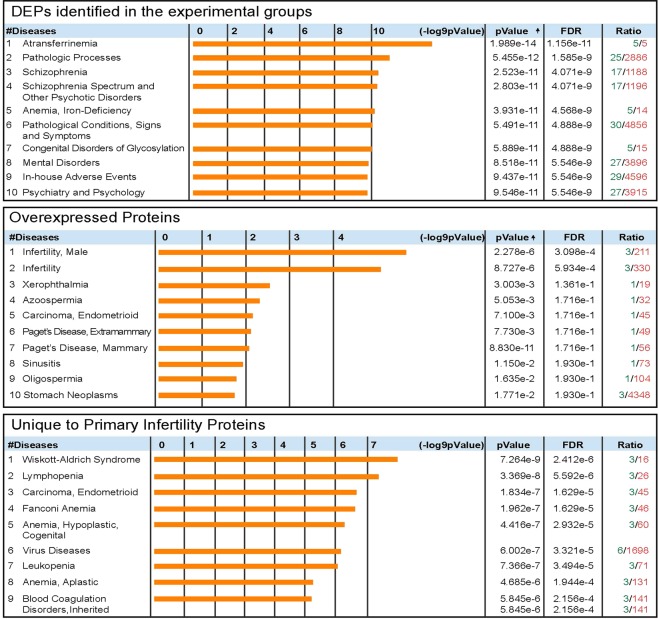
Diseases for differently expressed proteins and for overexpressed and unique proteins in seminal plasma of men with primary infertility compared with proven fertile group.

STRING analysis was performed to clarify the protein-protein interaction networks of DEPs identified in the seminal plasma of men with primary infertility (Fig. 2A). This online tool also enabled the identification of biological processes more relevant in the network based on Gene Ontology (GO). In the seminal plasma of men with primary infertility, we identified regulated exocytosis (GO:0045055) (False discovery rate (FDR) = 1.09e^−6^) and secretion by the cell (GO:0032940) (FDR = 1.09e^−6^) as the two most important biological processes. Other biological processes identified are listed in Fig. 2A. Using DAVID we were able to identify functional annotations for DEPs identified in seminal plasma of men with primary infertility. Key processes and functions involved in overexpressed, underexpressed and DEPs unique to seminal plasma of men with primary infertility are presented in Table 5. Finally, the diseases related to DEPs from seminal plasma of men with primary infertility were identified using MetaCore enrichment (Fig. 3).

**Table 5:** DAVID functional annotations for differently expressed proteins identified in seminal plasma.

Men with primary infertility compared with the control group	
**Key Processes (# of proteins)**	
Overexpressed Proteins	Cell migration (5), Oxidation-reduction process (5), Cell adhesion (4), Response to drug (5), Carbohydrate metabolic process (4), ECM organization (4), Angiogenesis (3)
Underexpressed Proteins	Coagulation (2), Positive regulation of serine-type endopeptidase activity (2), Negative regulation of sperm motility (2), Protein heterooligomerization (2)
Proteins Unique to Primary Infertility	Oxidation-reduction process (1), Immune response (1), Regulation of protein stability (1), Cell-cell adhesion (2)
**Key Functions (# of proteins)**	
Overexpressed Proteins	Calcium ion binding (6), Identical protein binding (6), Structural molecule activity (5), Myosin V binding (3), GDP binding (3), Structural constituent of cytoskeleton (3)
Underexpressed Proteins	Protease binding (2)
Proteins Unique to Primary Infertility	GTPase activity (1), Protein binding (3), Iron-ion binding (1), GTPase inhibitor activity (1)
**Men with secondary infertility compared with the control group**	
**Key Processes (# of proteins)**	
Overexpressed Proteins	Platelet degranulation (6), Negative regulation of endopeptidase activity (5), Carbohydrate metabolic process (5), Proteolysis (6), Cell-cell adhesion (4), PKA signaling (2)
Underexpressed Proteins	Coagulation (2), Negative regulation of sperm motility (2), Positive regulation of serine-type endopeptidase activity (2), antibacterial humoral response (20, protein heterooligomerization (2)
Proteins Unique to Secondary Infertility	ECM organization (3), Protein refolding (2), Cellular iron ion homeostasis (2), Actin cytoskeleton reorganization (2), Negative regulation of endopeptidase activity (2)
**Key Functions (# of proteins)**	
Overexpressed Proteins	Cadherin binding involved in cell-cell adhesion (6), Endopeptidase inhibitor activity (3), GTPase activity (4), Unfolded protein binding (3), GTP binding (4), Structural molecule activity (3), Calcium-dependent protein binding (3)
Proteins Unique to Secondary Infertility	Ferroxidase activity (2), Serine-type endopeptidase inhibitor activity (2), Unfolded protein binding (2), Identical protein binding (3)

**Figure 4:**
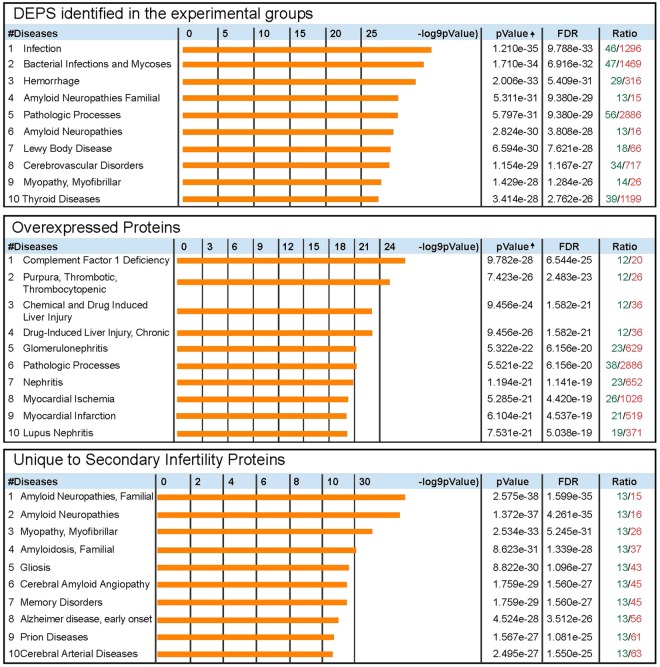
Diseases for differently expressed proteins and for overexpressed and unique proteins in seminal plasma of men with secondary infertility compared with proven fertile group.

String analysis was performed with the DEPs identified in men with secondary infertility (Fig. 2B). In the seminal plasma of men with secondary infertility, regulated exocytosis (GO:0045055) (FDR = 1.09e^−12^) and cell activation involved in immune response (GO:0002263) (FDR = 2.81e^−12^) were the two most important biological processes identified. Other recognized biological processes are presented in Fig. 2B. Using DAVID, we identified key processes and functions involved in overexpressed, underexpressed and DEPs unique to seminal plasma of men with secondary infertility (Table 5). Using MetaCore enrichment, the diseases related with DEPs from seminal plasma of men with secondary infertility were identified and presented in Fig. 4.

### Western blot validation of seminal plasma proteins

Based on selection criteria and the biological role, six proteins (ANXA2, PRDX2, CDC42, CD63, SEMG1 and SEMG2) from men with primary infertility and five proteins (ANXA2, C4, APP, SEMG1 and SEMG2) from men with secondary infertility were validated by WB.

**Figure 5:**
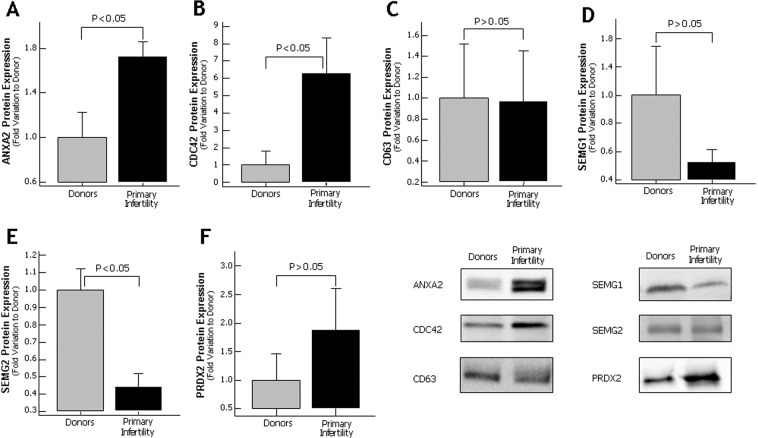
Protein expression levels of the differentially expressed proteins selected for validation by Western blot in seminal plasma of proven fertile donors’ group and with primary infertility. (**A**) Annexin A2; (**B**) CDC42 protein; (**C**) CD63; (**D**) Semenogelin 1 (SEMG1); (**E**) Semenogelin 2 (SEMG2) and (**F**) Peroxiredoxin 2; Results are expressed as mean ± SEM and in fold variation to donors’ group. Panel shows a representative image of Western Blot experiments.

**Figure 6:**
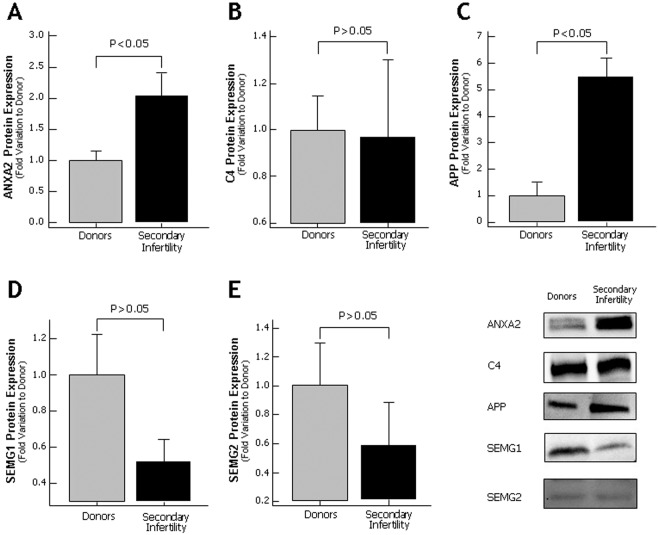
Protein expression levels of the differentially expressed proteins selected for validation by Western blot in seminal plasma of proven fertile donors’ group and with secondary infertility. (**A**) Annexin A2; (**B**) C4 protein; (**C**) Amyloid precursor protein (APP); (**D**) Semenogelin 1 (SEMG1) and (**E**) Semenogelin 2 (SEMG2). Results are expressed as mean ± SEM and in fold variation to donors’ group. Panel shows a representative image of Western Blot experiments.

Of the proteins analyzed in men with primary infertility, ANXA2 showed an increased expression when compared to fertile donors (p < 0.05) (Fig. 5A). Other three proteins (PRDX2, CD63 and SEMG1) were detected by WB, but no differences were observed in their expression levels compared to control group (Fig. 5C,D,F). The WB validation of the protein CDC42 showed an increase in protein expression (p < 0.05) (Fig. 5B) that was in concordance with the proteomic results. The other protein SEMG2 selected for validation by western blot was underexpressed in seminal plasma of men with primary infertility when compared to fertile donors (p < 0.05) (Fig. 5E). The protein C4, SEMG1 and SEMG2 selected for validation using western blot in secondary infertility group did not show any change in the expression (Fig. 6B,D,E). Other key proteins such as ANXA2 and APP were overexpressed in secondary infertility group compared with control group (p < 0.05) (Fig. 6A,C).

## Discussion

The global proteomic approach is currently being used to identify the molecular factors associated with the cause of male infertility. As spermatozoa are transcriptionally and translationally silent they depend on the sperm and the seminal plasma proteins for their normal biological functions. Seminal plasma harbors factors that are essential for protection of spermatozoa during its transit through female reproductive tract and assist in the fertilization process. Semen analysis of men with primary or secondary infertility revealed that all semen parameters were above the WHO 2010 reference values^39^ (Table 2). Since semen quality seems not to be the cause for infertility in these men, we used proteomic approach to identify the dysregulated seminal plasma proteins. Intasqui *et al*. carried out proteomic profiling of sperm in men with primary and secondary infertility, and proposed BAG6 and HIST1H2BA as potential biomarker of male infertility^40^. As a follow up, the current study focused on the proteomic analysis of seminal plasma and its bioinformatic analysis that provides an extensive information about distribution, molecular and functional analysis of the identified proteins^9,11,38,41^. Furthermore, the proteins selected based on reproductive functions and fertilization process such as regulation of exocytosis^42,43^, regulation of cell motility^44,45^ or vesicle mediated-transport^46^ were validated using WB technique. These biological processes are crucial for fertilization and our analysis revealed dysregulation in both men with primary and secondary infertility. Role of key proteins associated with the pathophysiology of primary and secondary male infertility are discussed in detail.

Annexin 2 (ANXA2) is a Ca^2+^-dependent phospholipid-binding protein, which is associated with plasma membrane of cells and endosomes^47^. ANXA2 plays an important role in cellular processes, such as, membrane trafficking events^48^, lipid reorganization in the membrane and endocytosis^49^. During abnormal ubiquitination process, an aberrant expression of ANXA2 was observed^50,51^. With respect to reproductive functions, ANXA2 is involved in maintaining the integrity of blood-testis-barrier and in the release of spermatozoa^52^. Presence of ANXA2 in sperm is essential for the binding of the sperm in the female tract that is crucial for fertilization^53^. Earlier studies have reported abnormal expression of ANXA2 in seminal plasma and prostasomes of subfertile or infertile men^54–56^. In the current study, ANXA2 was found to be involved in several biological function such as exocytosis and secretion by cell, both processes essential in the binding to sperm. Proteomic analysis revealed overexpression of ANXA2 in the seminal plasma of men with primary and secondary infertility. Furthermore, WB findings also confirmed the overexpression of ANXA2 in both primary and secondary infertile men compared to control group. Hence, we suggest that aberrant expression of ANXA2 in the seminal plasma affects the maturation process of the spermatozoa^55^, resulting in production of the immature sperm in both primary and secondary male infertility conditions.

Peroxiredoxins (PRDXs) are the key proteins that regulate ROS levels and plays an important role in male fertility^57^. Bioinformatic analysis of DEPs present in the seminal plasma of primary infertility men confirmed that PRDX2 was involved in the oxidation-reduction process. In spermatozoa, the presence of PRDX2 was detected in plasma membrane, acrosome, nucleus, midpiece and flagellum^58^. PRDX2 reduces the availability of iron which in turn decreases oxidative stress^59^. Alteration in the expression of this protein has been reported in men with immunological infertility^60^. Proteomic analysis of seminal plasma of men with primary infertility revealed overexpression PRDX2, whereas WB validation did not reveal difference in the expression levels of PRDX2 compared to the control group. The discrepancies in the results of LC-MS/MS and WB could be mainly due to the differences in the specificity and sensitivity of both techniques. Our seminal plasma proteomic results are concurrent with the findings from earlier reports on male infertility conditions related to varicocele and in men with Hodgkin’s disease^61,62^. Increased levels of PRDX2 in the seminal plasma of men with primary infertility indicates oxidative stress mediated damage to the spermatozoa leading to impairment of physiological functions related to fertilization process such as hyperactivation, capacitation, acrosome reaction and sperm-oocyte fusion.

The protein CD63 antigen (CD63) is a cellular trafficking molecule^63^ and is considered an exosomal marker^64^. Dysregulation in the expression of CD63 affects the exosome-sperm fusion, which is responsible for the production of immature spermatozoa^65^. In the current study, CD63 was overexpressed in primary infertility group and was involved in vesicle-mediated transport. Alteration in the expression of CD63 protein was reported in spermatozoa of men with testicular cancer^66^ and seminal plasma of infertile varicocele men^55^. Furthermore, our bioinformatic analysis revealed that the overexpressed seminal plasma protein CDC42 was also involved in vesicle-mediated transport. CDC42 proteins are expressed in the head of elongated spermatids^67^ and are involved in the formation of sperm tail and head^68^. CDC42 are identified as key controllers of capacitation-dependent actin dynamics^69^ and regulators of the acrosome reaction^70^. Dube *et al*. reported decreased levels of CDC42 transcripts in obstructive azoospermia^71^. Increase levels of CDC42 and CD63 in seminal plasma may play a major role in the pathology associated with vesicle-mediated transport in men with primary infertility. Further, CD63 has a direct impact on the transport of exosomal molecules/ factors to the spermatozoa required for the maturation process whereas CDC42 protein dysfunction may serve as factor for compromised acrosome reaction in men with primary infertility. We were also able to demonstrate the presence of both the proteins (CDC42 and CD63) using WB in the seminal plasma of men with primary infertility.

Immune response and inflammation have negative impact on the male reproductive system and male fertility^72^. LC-MS/MS analysis and further confirmation by WB, revealed that C4 protein, which is involved in body’s immune response^73^, was overexpressed in secondary infertile men. Further, bioinformatics analysis also predicted infection as the most relevant dysregulated pathway in secondary infertility. Likewise, the multifunctional protein APP, which was detected in the seminal plasma of secondary infertility men, is involved in many biological processes such as exocytosis, cell activation to response of immune system, proteolysis regulation and regulation of iron homeostasis. Earlier studies demonstrated the localization of APP in tail and head of spermatozoa^74,75^. In oxidative stress mediated male infertility aberrant expression of APP results in acrosome dysfunction^76^. In the current study APP was overexpressed and described to have a role in sperm motility and interaction of the sperm with the oocyte^75^. Moreover, the validation of proteomic results using WB suggests that APP can serve as a seminal plasma marker in diagnosis of secondary infertility. Hence, from the global proteomic and WB results it is clear that proteins associated with immune response are overexpressed in the seminal plasma of men with secondary infertility and thus can a predisposing cause for this condition.

The most abundant proteins in seminal plasma are the semenogelins (1 and 2)^77^. These proteins are secreted by the seminal vesicles and form the gel-like coagulum with the fibronectin in the ejaculated semen^78^. The coagulum is liquefied through the action of prostate specific antigen protein, responsible for semenogelins cleavage, inducing motility and releasing spermatozoa. de Lamirande *et al*. reported that SEMGs are involved in the inhibition of capacitation process of human spermatozoa^79^. Other global proteomic studies also demonstrated the abnormal expression of SEMGs in seminal plasma of men with abnormal semen parameters^80^, seminal oxidative stress^81^ and varicocele^82,83^. In the present study, proteomic profile of seminal plasma showed both SEMG1 and SEMG2 were underexpressed in primary and secondary infertile men compared to control group. Dysregulation of the semenogelins in both primary and secondary infertility condition may have a severe impact on the capacitation process of the spermatozoa. Furthermore, validation of SEMG2 using WB in the seminal plasma of men with primary infertility suggests that SEMG2 levels can serve as candidate marker to differentiate primary from secondary infertility.

## Conclusion

The proteomic data from this pilot study shows an altered seminal plasma proteome in primary and secondary infertility compared with fertile men. Our preliminary results have identified maturation failure and immune reaction response as the causes for infertility in men with primary and secondary infertility. Validation of key proteins suggests that ANXA2 can be a screening biomarker for both primary and secondary infertility, whereas CDC42 and SEMG2 can be useful candidate biomarkers for primary infertility and APP for secondary infertility. This is the first report showing DEPs in seminal plasma of men with primary and secondary infertility, however further studies are warranted to confirm and validate these biomarkers.

## Supplementary information

Supplementary information.
