# Author Correction: Alterations in seminal plasma proteomic profile in men with primary and secondary infertility

**DOI:** 10.1038/s41598-020-69838-7

**Published:** 2020-08-04

**Authors:** Ana D. Martins, Manesh Kumar Panner Selvam, Ashok Agarwal, Marco G. Alves, Saradha Baskaran

**Affiliations:** 1 American Center for Reproductive Medicine, Cleveland Clinic, Cleveland, OH USA; 2 Department of Microscopy, Laboratory of Cell Biology, Institute of Biomedical Sciences, Abel Salazar and Unit for Multidisciplinary Research in Biomedicine, University of Porto, Porto, Portugal

Correction to: *Scientific Reports*10.1038/s41598-020-64434-1, published online 05 May 2020

In Figure 6, the blot for SEMG1 is duplicated from Figure 5. The correct Figure 6 appears below as Figure 1.

**Figure 1:**
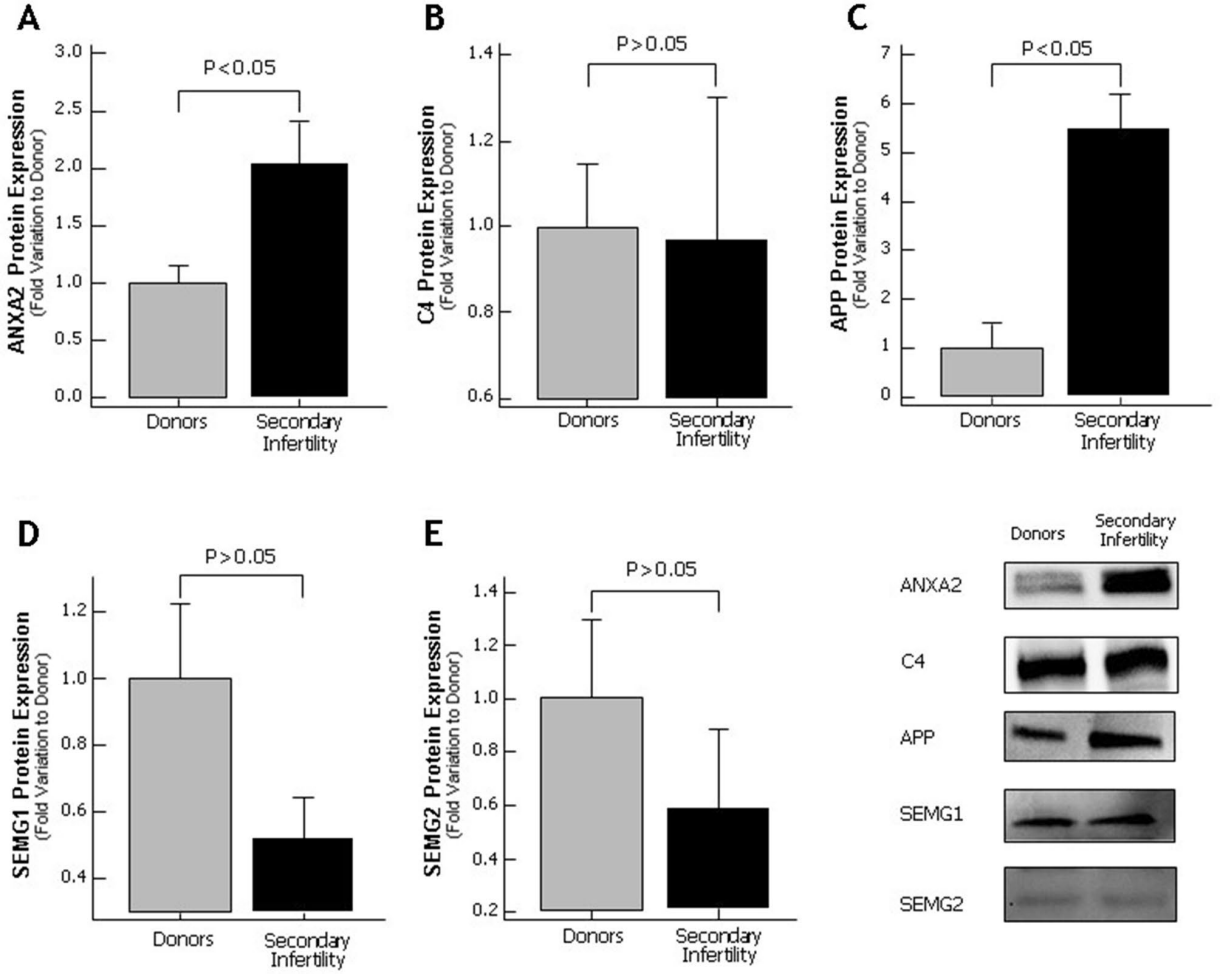
Protein expression levels of the differentially expressed proteins selected for validation by Western blot in seminal plasma of proven fertile donors’ group and with secondary infertility. (**A**) Annexin A2; (**B**) C4 protein; (**C**) Amyloid precursor protein (APP); (**D**) Semenogelin 1 (SEMG1) and (**E**) Semenogelin 2 (SEMG2). Results are expressed as mean ± SEM and in fold variation to donors’ group. Panel shows a representative image of Western Blot experiments.

Additionally, the Supplementary Information file that accompanies this Article contains an error, where Supplementary Figure S10 is a duplicate of Supplementary Figure S4. The correct Supplementary Figure S10 appears below as Figure 2.

**Figure 2:**
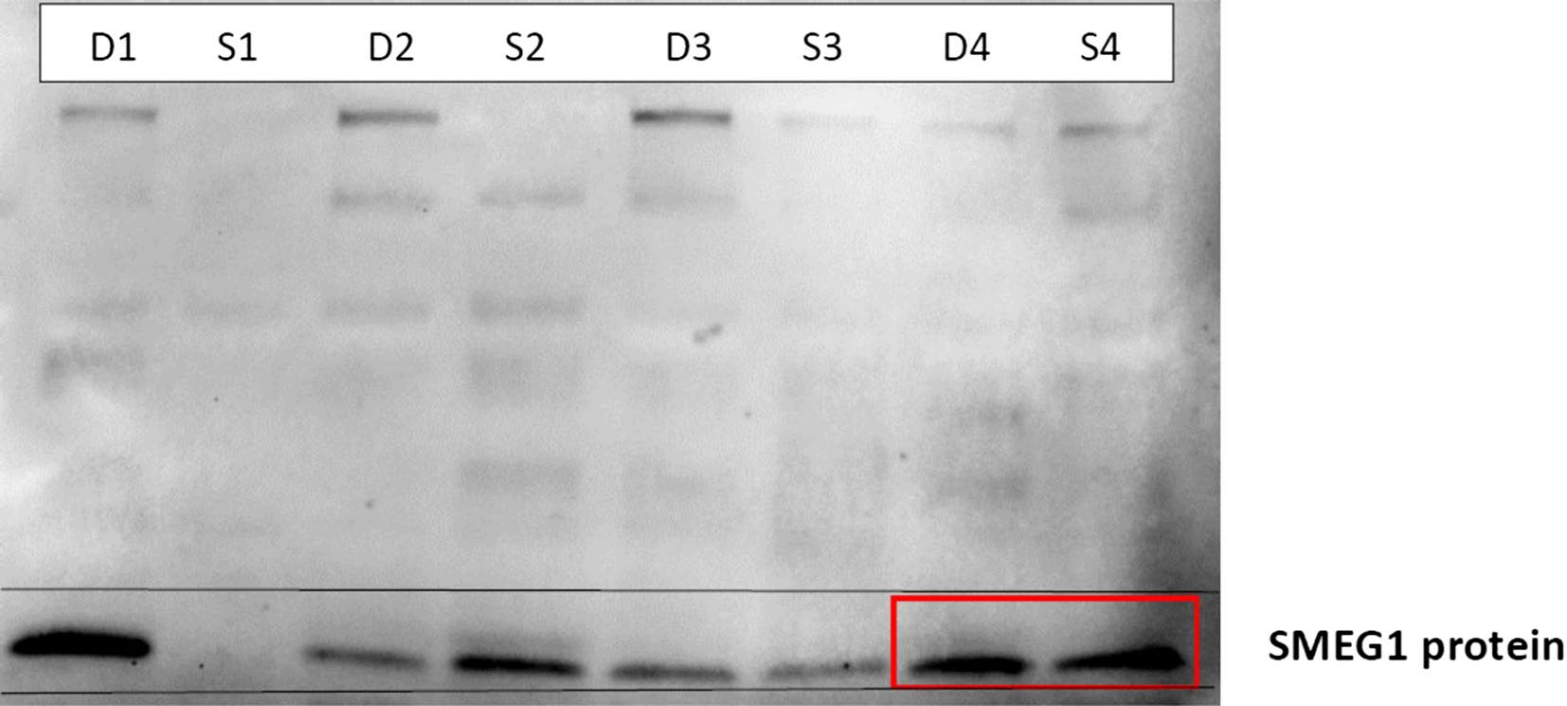
Western blot of SEMG1 in seminal plasma of proven fertile donors’ group and with secondary infertility.

