# Supplementary material for: Alterations in seminal plasma proteomic profile in men with primary and secondary infertility
Source: Sci Rep. 2020 May 5;10:7539. doi: 10.1038/s41598-020-64434-1 (PMC7200760; doi:10.1038/s41598-020-64434-1)

Supplementary File

Alterations in seminal plasma proteomic profile in men with primary and secondary infertility

[1]Ana D. Martins, [1]Manesh Kumar Panner Selvam, *[1]Ashok Agarwal, [2]Marco G. Alves, [1]Saradha Baskaran

[1]American Center for Reproductive Medicine, Cleveland Clinic, Cleveland, OH, USA.

[2]Department of Microscopy, Laboratory of Cell Biology, Institute of Biomedical Sciences Abel Salazar and Unit for Multidisciplinary Research in Biomedicine, University of Porto, Porto, Portugal.

Figure S1: Western blot of ANXA2 in seminal plasma of proven fertile donors' group and with primary infertility.

Figure S2: Western blot of CDC42 in seminal plasma of proven fertile donors' group and with primary infertility.

Figure S3: Western blot of CD63 in seminal plasma of proven fertile donors' group and with primary infertility.

Figure S4: Western blot of PRDX2 in seminal plasma of proven fertile donors' group and with primary infertility.

Figure S5: Western blot of SEMG1 in seminal plasma of proven fertile donors' group and with primary infertility.

Figure S6: Western blot of SEMG2 in seminal plasma of proven fertile donors' group and with primary infertility.

Figure S7: Western blot of ANXA2 in seminal plasma of proven fertile donors' group and with secondary infertility.

Figure S8: Western blot of C4 protein in seminal plasma of proven fertile donors' group and with secondary infertility.

Figure S9: Western blot of APP in seminal plasma of proven fertile donors' group and with secondary infertility.

Figure S10: Western blot of SEMG1 in seminal plasma of proven fertile donors' group and with secondary infertility.

Figure S11: Western blot of SEMG2 in seminal plasma of proven fertile donors' group and with secondary infertility.

Figure S1:

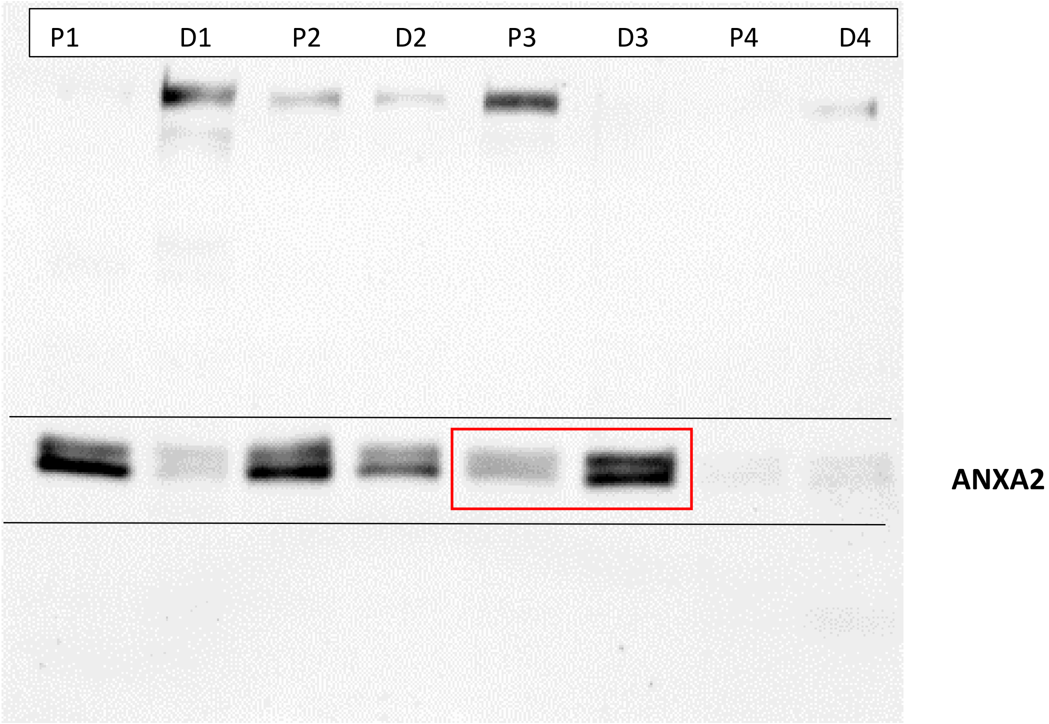

Figure S2:

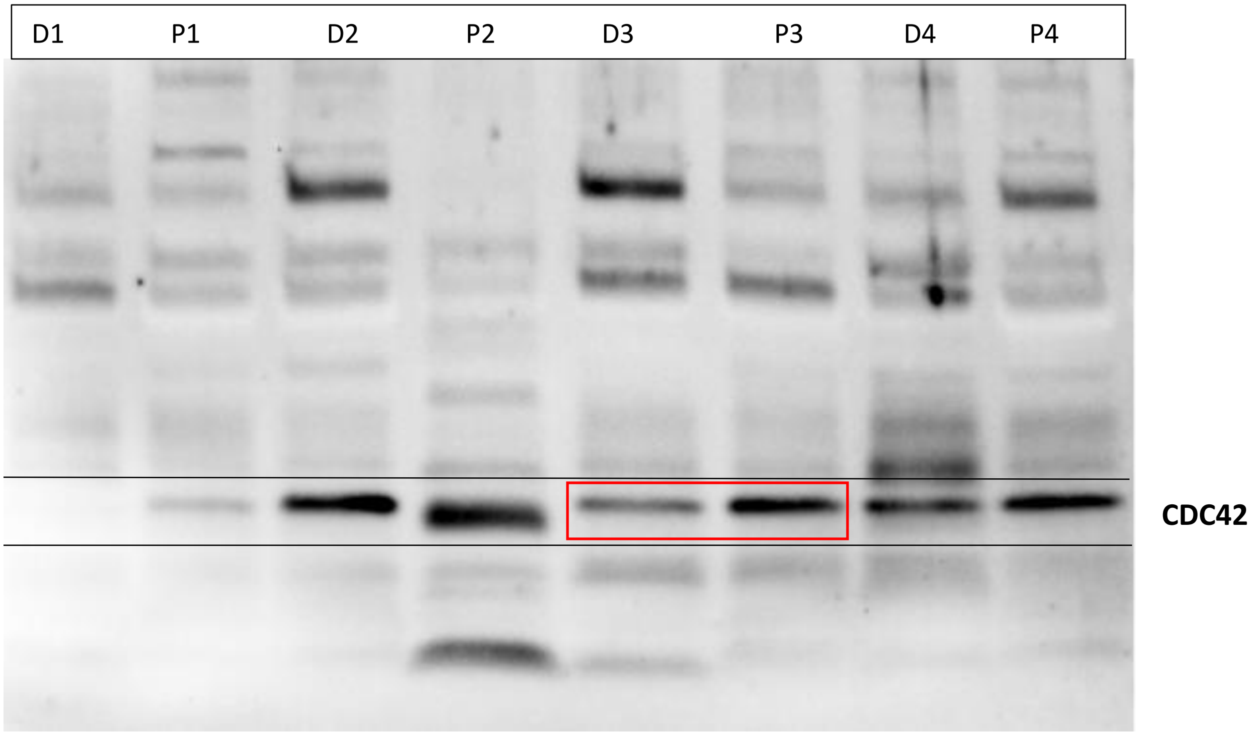

Figure S3:

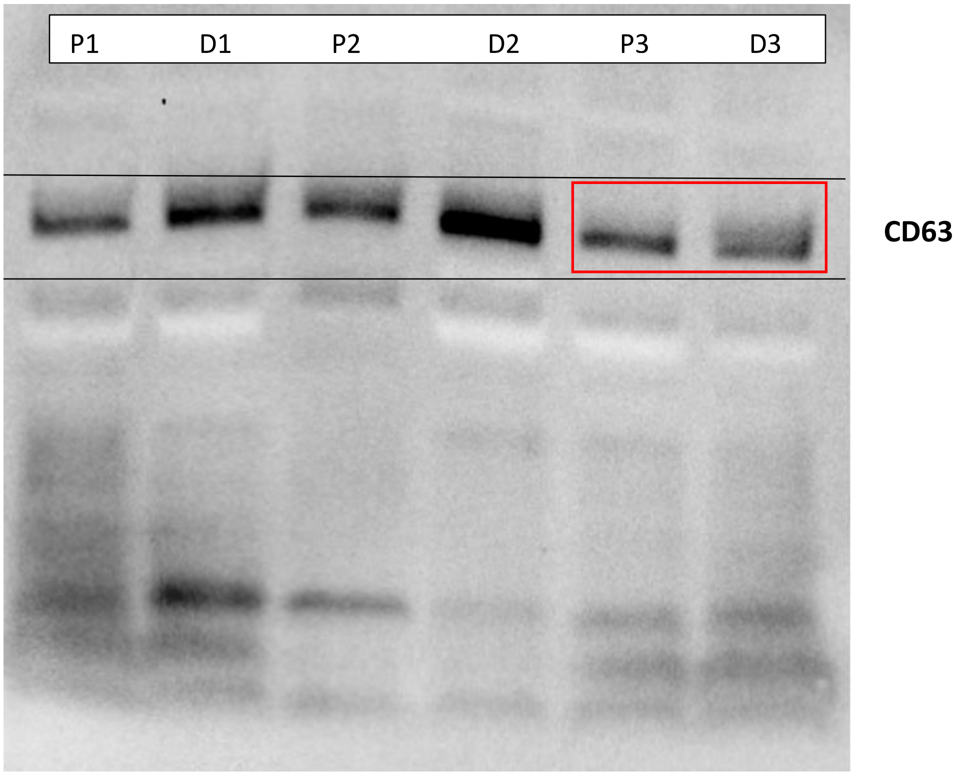

Figure S4:

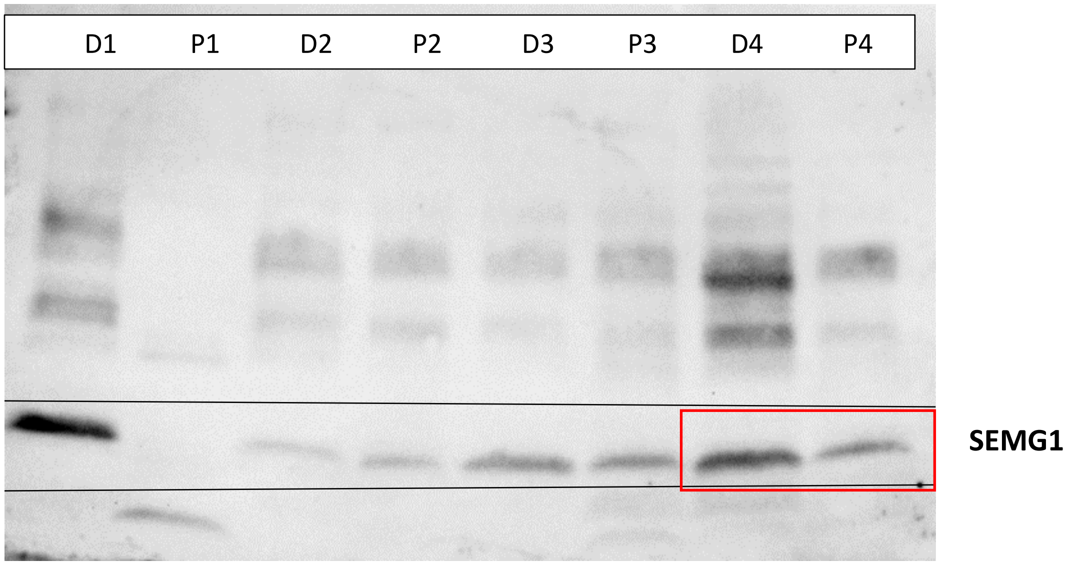

Figure S5:

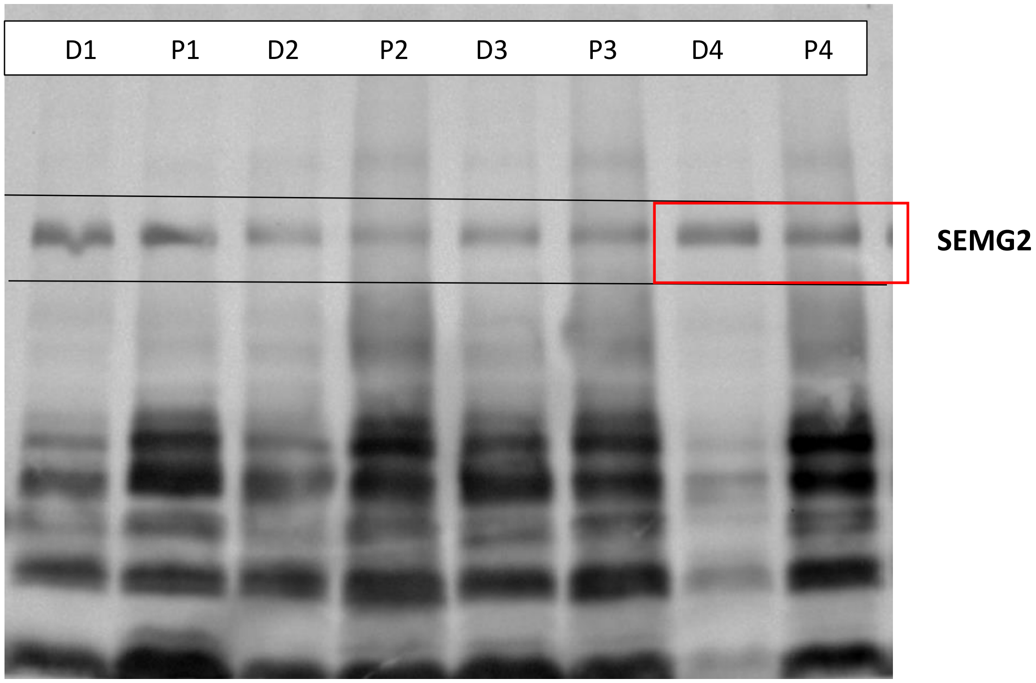

Figure S6:

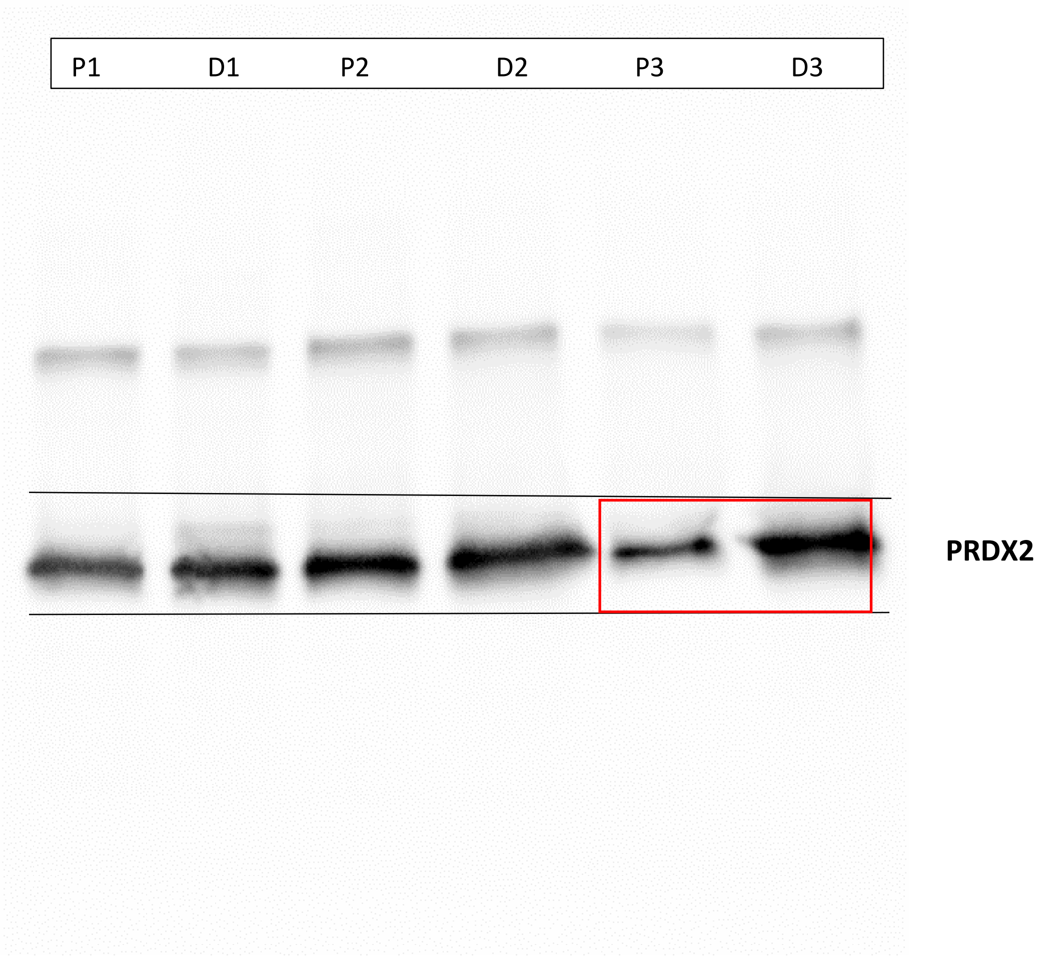

Figure S7:

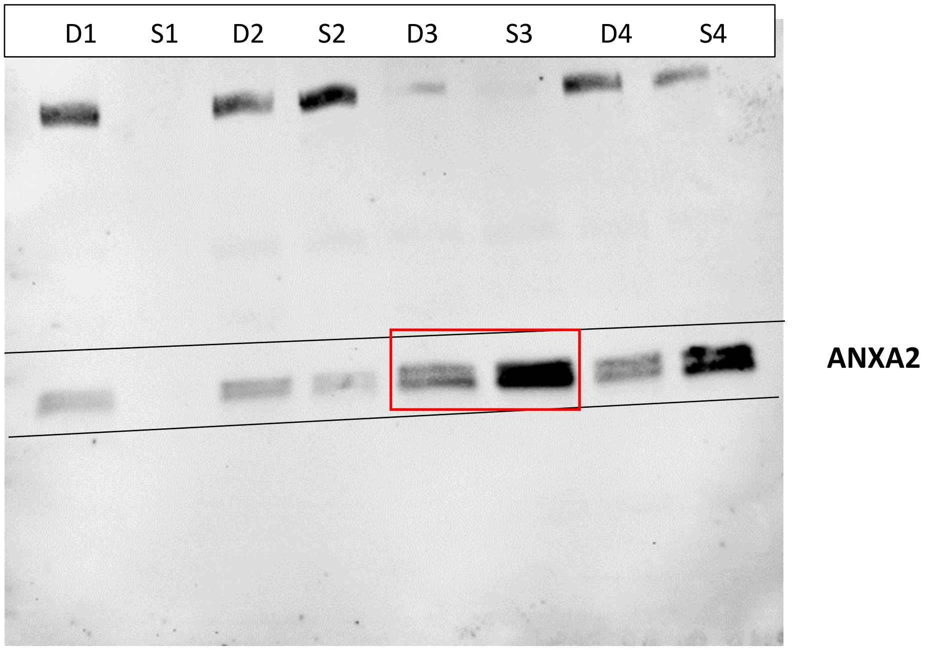

Figure S8:

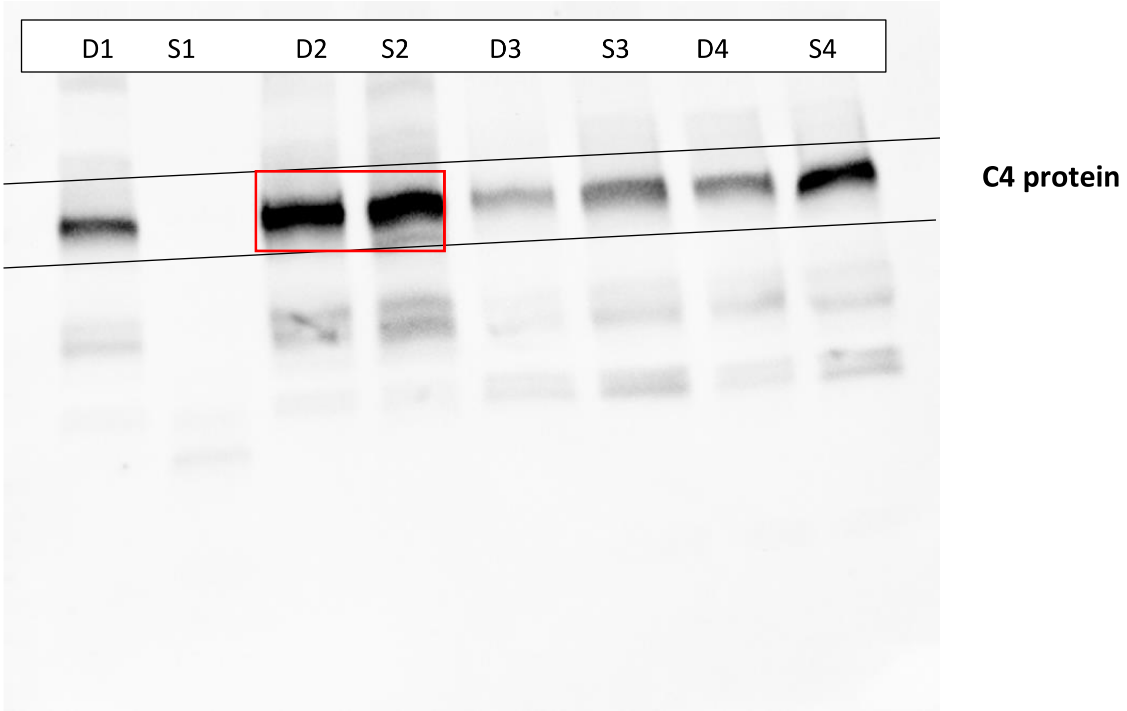

Figure S9:

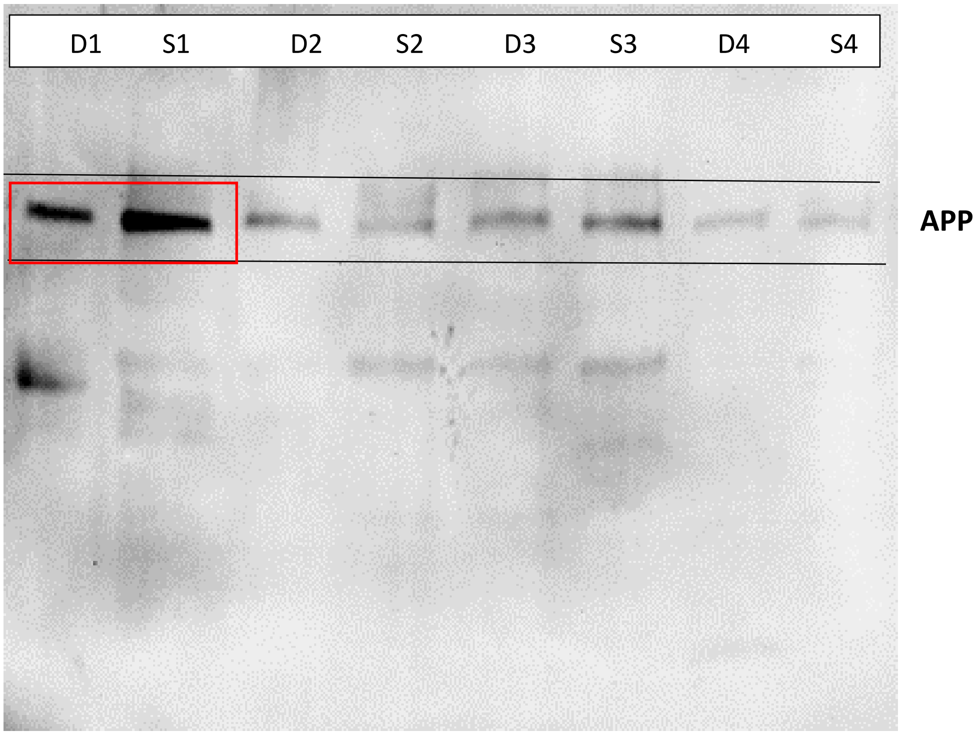

Figure S10:

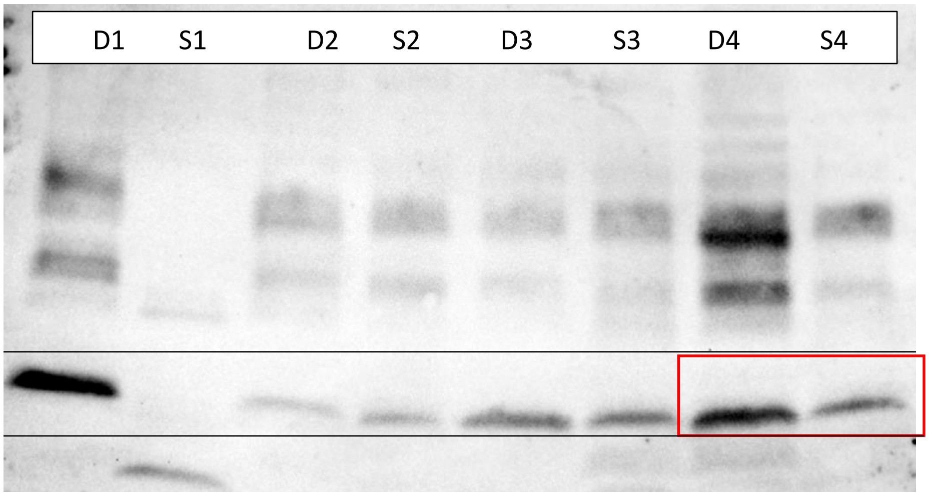

SMEG1 protein

Figure S11:

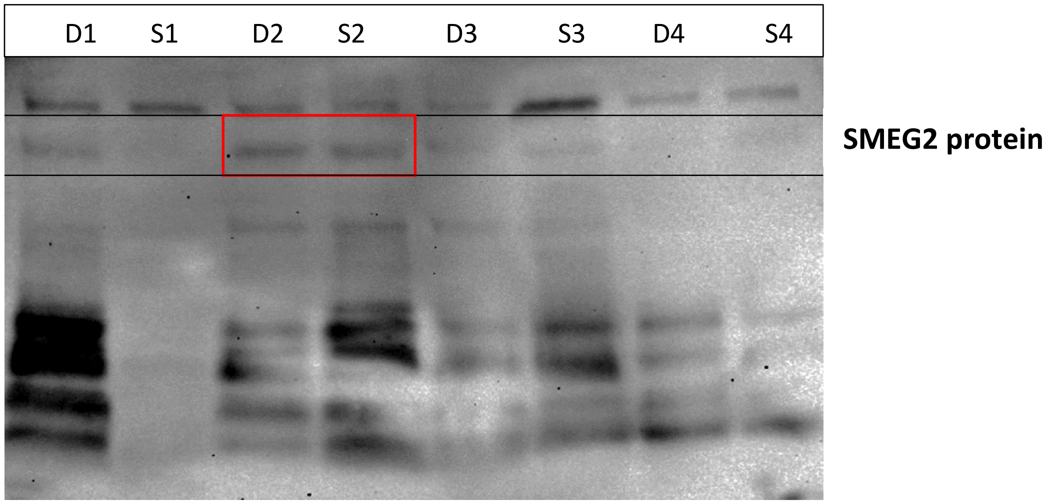

Supplement: Supplementary file 1 — Supplementary information. [file 41598_2020_64434_MOESM1_ESM.pdf]
